# Case Report: Synchronous cervical, gastric and rectal metastases in occult breast cancer

**DOI:** 10.3389/fonc.2025.1598840

**Published:** 2025-09-08

**Authors:** Ruoying Deng, Ze Meng, Qiang Li, Yarong Wang, Yuyun Lan, Yao Liu, Yibing Liu

**Affiliations:** ^1^ Department of Oncology, The Fourth Hospital of Hebei Medical University, Shijiazhuang, Hebei, China; ^2^ Department of Digestive, The Second Affiliated Hospital of Xingtai Medical College, Xingtai, Hebei, China; ^3^ Department of Imaging, The Second Affiliated Hospital of Xingtai Medical College, Xingtai, Hebei, China

**Keywords:** occult primary breast cancer, simultaneous metastases, cervical, gastric and rectal metastasis, endocrine therapy, CDK4/6 inhibitors

## Abstract

Occult breast cancer (OBC) is characterized as a breast carcinoma that remains undetectable through imaging modalities. OBC is a rare condition, accounting for only 0.3%-1.0% of all breast cancers, and is often clinically associated with metastases to the axillary and cervical lymph nodes. Metastasis of occult primary breast cancer to the cervix, stomach, or rectum is exceptionally rare. Here, we report the case of a 64-year-old woman presenting with clinical symptoms of urinary urgency, frequency, severe rectal discomfort, and altered stool morphology. Ga68-positron emission tomography-computed tomography (Ga68-FAPI PET/CT) identified diffuse tumor-associated fibrin expression within the gastric wall, while 18F-fluorodeoxyglucose PET/CT (18F-FDG PET/CT) revealed marked annular thickening of the rectal wall. Notably, imaging evaluations, including mammography, breast ultrasonography, and 18F-FDG PET/CT, failed to detect primary breast lesions. Histopathological examination and immunohistochemical analysis of biopsied cervical, gastric, and rectal lesions confirmed the diagnosis of OBC. Following six months of treatment with letrozole (1mg/day) in combination with dalpiciclib the patient demonstrated significant symptomatic relief, with remarkable reduction in the lesions located in the cervix, stomach, and rectum. This case represents the first documented instance of occult primary breast cancer with simultaneous metastases to the cervix, stomach, and rectum.

## Introduction

Breast cancer is the most common malignancy among women and remains the leading cause of cancer-related mortality in women worldwide. In 2020, approximately 2.3 million new cases of breast cancer were reported globally, with more than half of these diagnoses occurring in low-and middle-income countries. That same year, breast cancer claimed the lives of 685,000 women (1). China bore the highest burden of breast cancer, with 357,200 newly diagnosed cases and 75,000 deaths reported in 2020 (2).

Occult breast cancer (OBC) is a rare clinical entity, defined as metastatic cancer originating from malignant primary breast tumors that remain undetectable on clinical examination and radiological imaging. OBC accounts for 0.3%-1.0% of all breast cancers and typically presents at a median age of approximately 55 years (3, 4). The diagnosis of OBC is often challenging due to its non-specific symptoms and the absence of detectable breast lesions. Axillary lymph node metastasis is the most common clinical presentation (5), although metastases to other sites, including the bone, liver, lymphatic system, skin, orbit, bone marrow, lung, spleen, and cervix, have also been reported (6–12). OBC predominantly affects older adults and is generally diagnosed at an early stage, often associated with a favorable prognosis. However, to date, no cases of simultaneous metastases of primary OBC to the cervix, stomach, and rectum have been reported in the literature. Here, we present a unique case of concurrent cervical, gastric, and rectal metastases in a patient with occult primary breast cancer. This report aims to provide insights into the diagnostic challenges and therapeutic approaches for this rare presentation.

## Case report

A 64-year-old postmenopausal Chinese woman with a 10-year history of hypertension, managed with regular oral antihypertensive medication, presented in May 2023 with complaints of urinary urgency, frequent urination, severe urgency, altered stool morphology, occasional abdominal distension, and abdominal pain. These symptoms were alleviated following initial treatment. Notably, there was no vaginal bleeding, fluid discharge, dysuria, or hematochezia. A review of her medical history revealed no significant findings, and she reported no history of smoking, alcohol consumption, illicit drug use, or a family history of cancer.

On physical examination, there was no palpable axillary, supraclavicular, or inguinal lymphadenopathy. However, a cervical mass was identified during anal examination, while endoscopy revealed an intra-anal annular mass. A gynecological examination further identified a frozen pelvis, with the cervix described as hyperplastic, firm, and morphologically fair. The vaginal vault and upper vaginal tissue on both sides were palpably firm and hardened.

**Figure 1:**
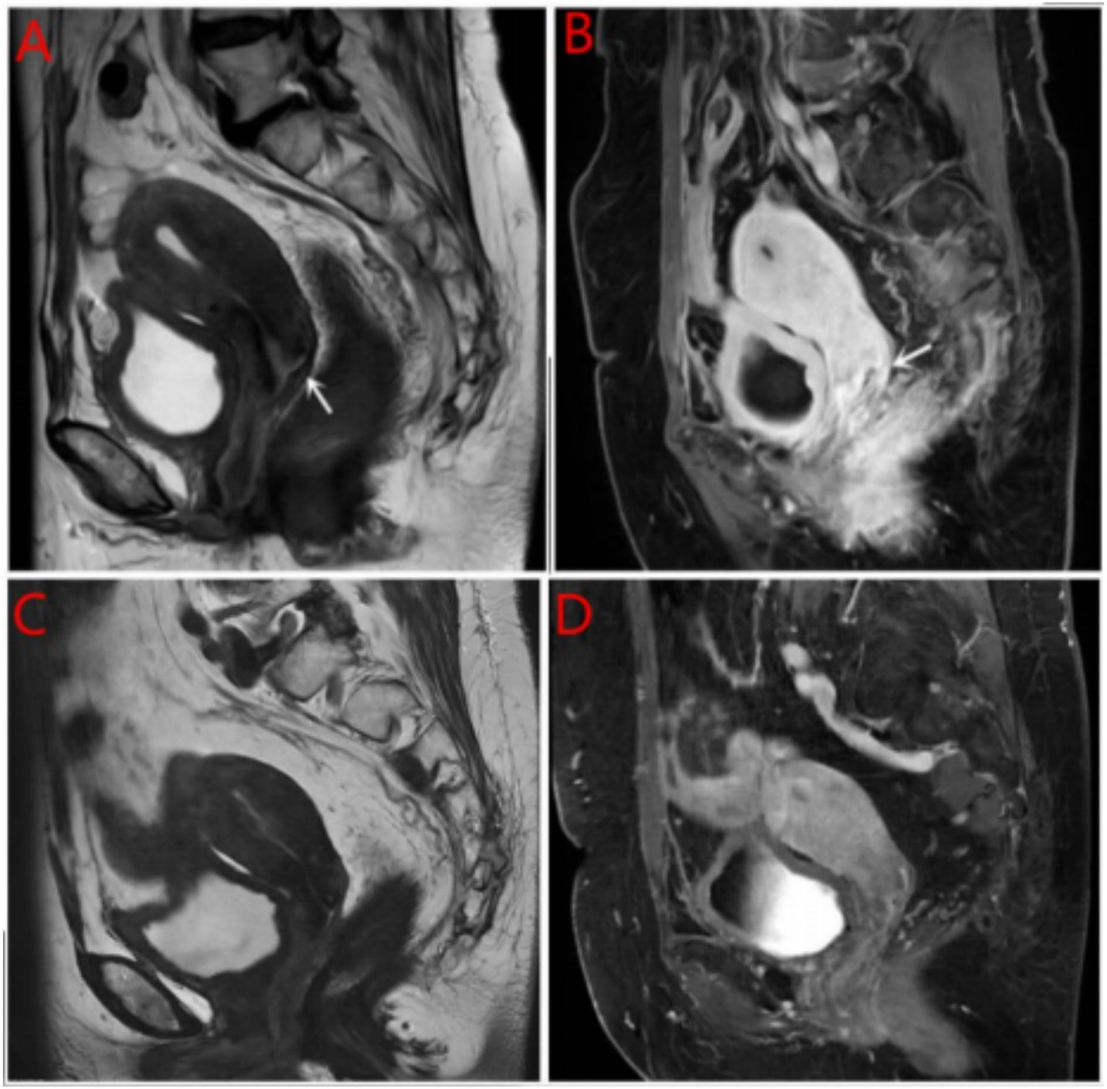
MRI cervical mass. **(A)** Pretreatment with MRI T2W1 imaging of the cervical mass; **(B)** Pretreatment with enhanced MRI imaging of the cervical mass; **(C)** Post treatment with MRI T2W1 imaging of the cervical mass; **(D)** Post treatment with enhanced MRI imaging of the cervical mass.

**Figure 2:**
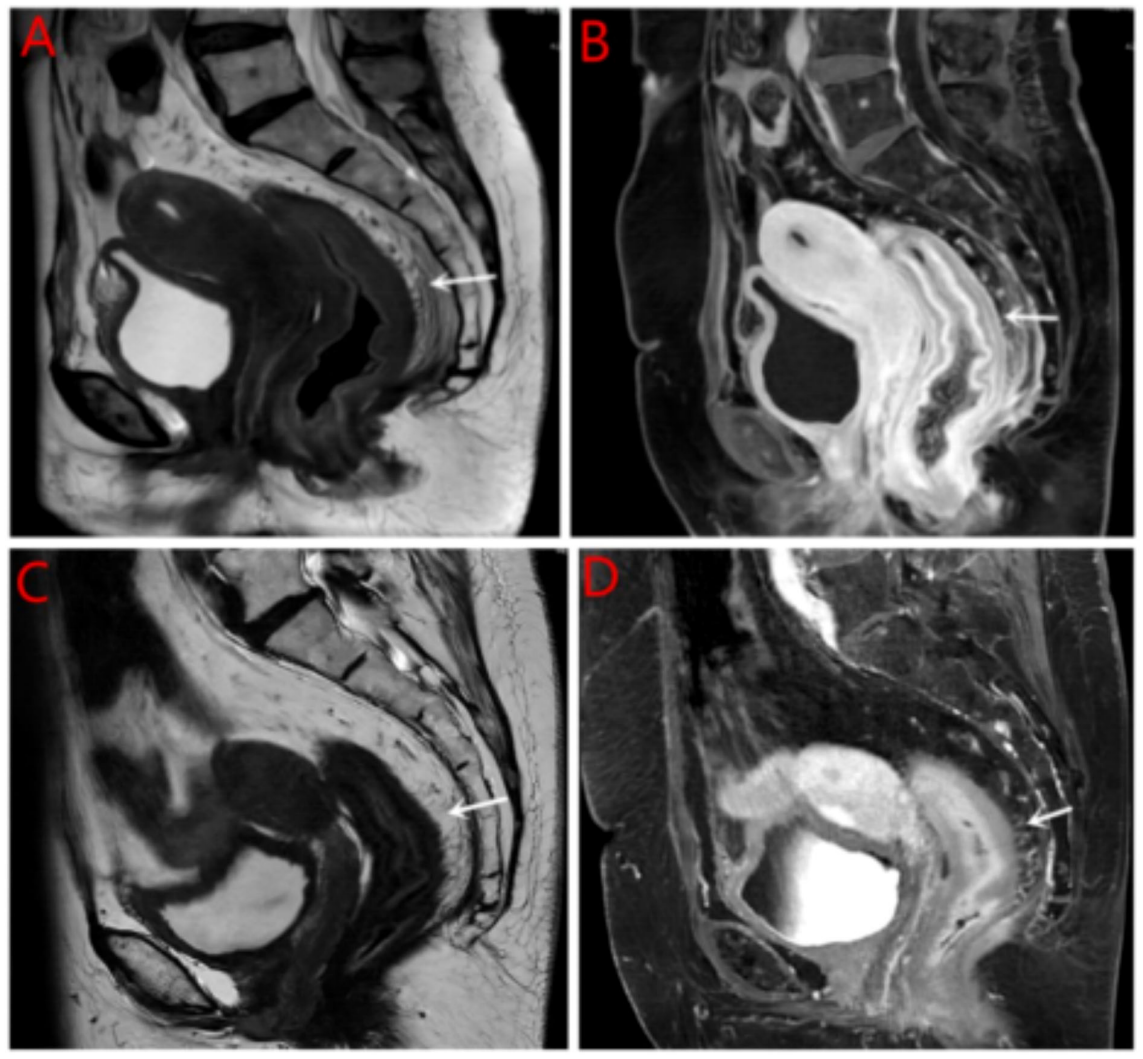
MRI rectal wall. **(A)** Pretreatment with MRI T2W1 imaging of the rectal wall; **(B)** Pretreatment with enhanced MRI imaging of the rectal wall; **(C)** Post treatment with MRI imaging of the rectal wall; **(D)** Pretreatment with enhanced MRI imaging of the rectal wall.

**Figure 3:**
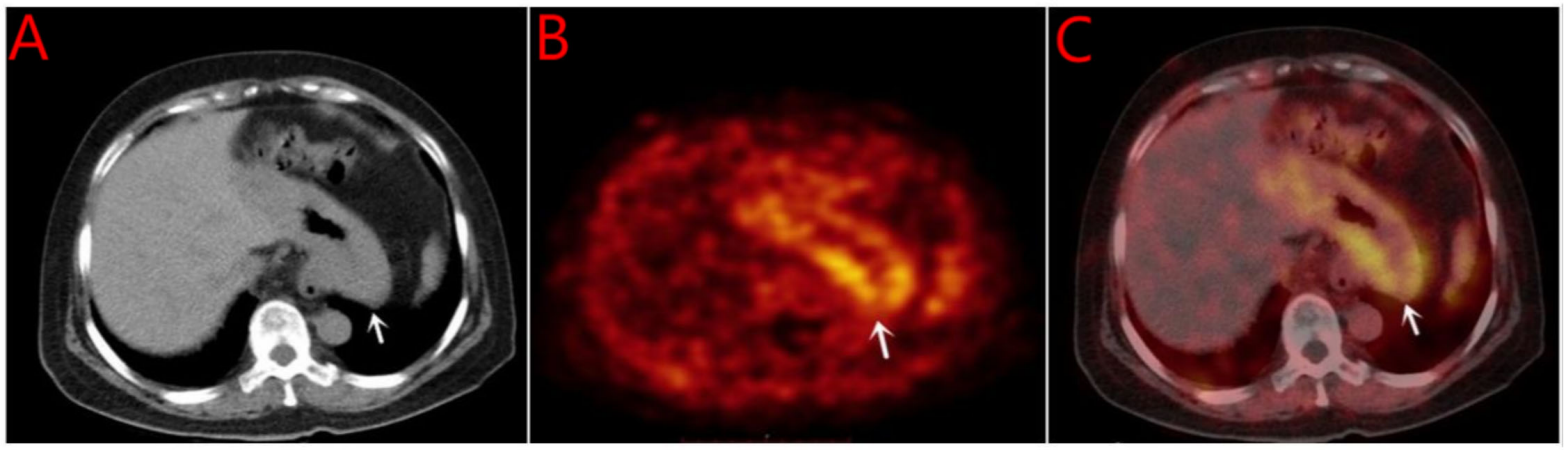
Ga68-FAPI PET/CT before gastric treatment. **(A)** CT shows thickening of gastric wall; **(B)** PET shows high metabolism; **(C)** MIP shows of gastric wall. Ga68-FAPI PET/CT, G68 positron emission tomography-computed tomography; MIP, Maximum Intensity Projection.

**Figure 4:**
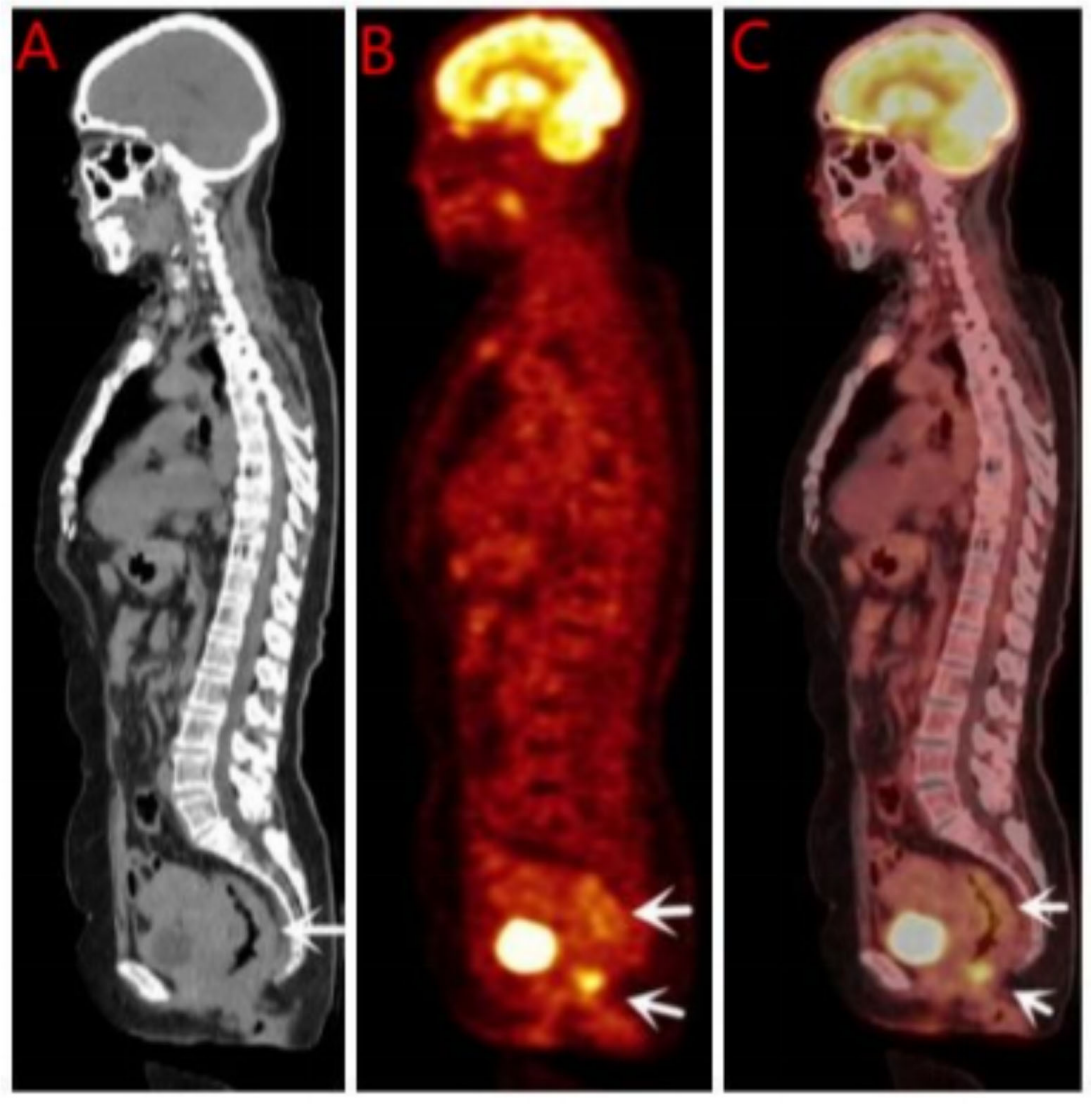
18F-FDG PET/CT before rectal treatment. **(A)** CT shows thickening of rectal wall; **(B)** PET shows high metabolism; **(C)** MIP shows of rectal wall. 18F-FDG PET/CT, 18F-flurodeoxyglucose positron emission tomography-computed tomography; MIP, Maximum Intensity Projection.

**Figure 5:**
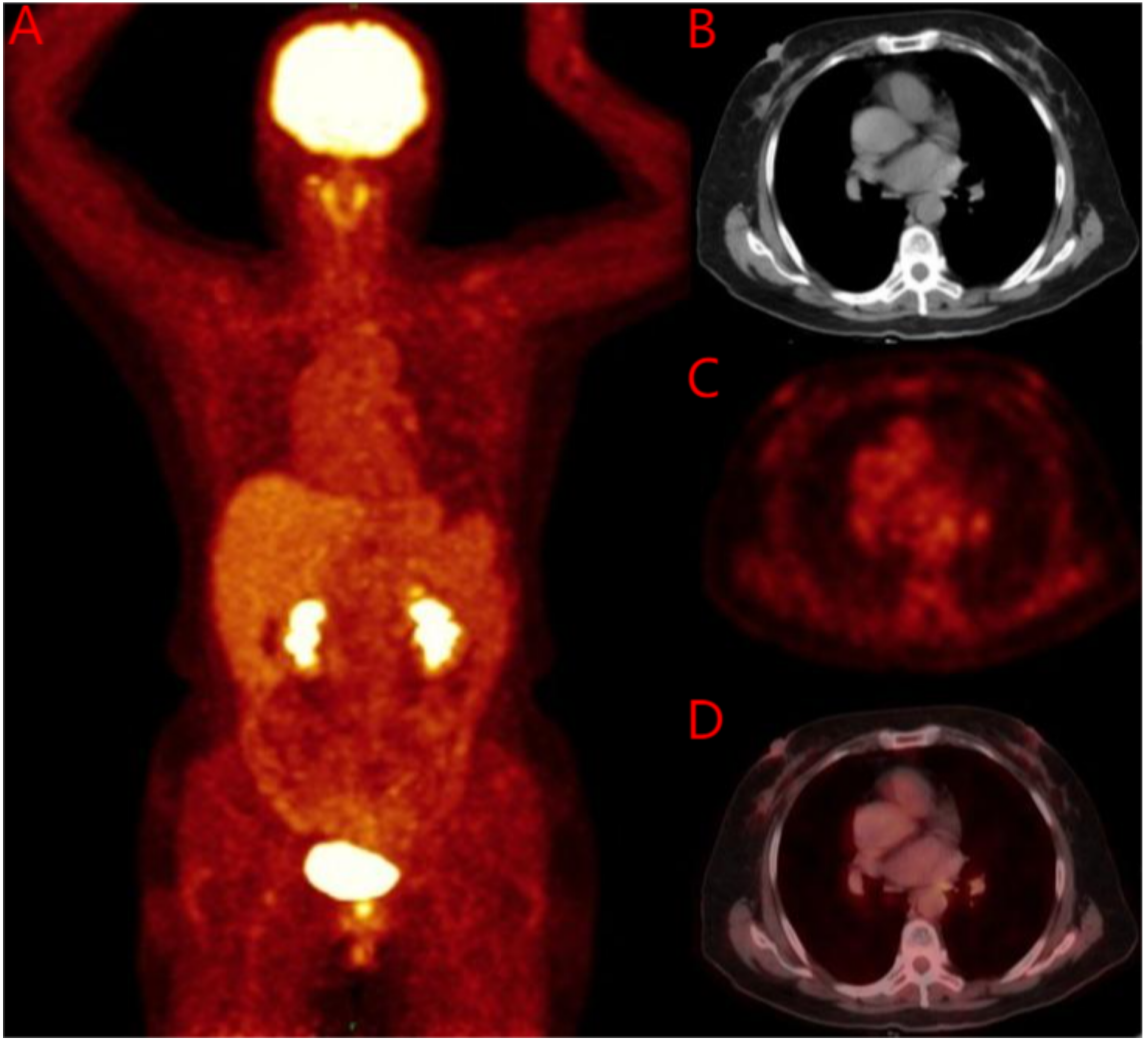
18F-FDG PET/CT before breast treatment. **(A)** Whole body image of 18F-FDG PET/CT; **(B)** No soft tissue mass was found on breast CT; **(C)** No soft tissue mass was found on breast PET; **(D)** No soft tissue mass was found on breast MIP. 18F-FDG PET/CT, 18F-flurodeoxyglucose positron emission tomography-computed tomography; MIP, Maximum Intensity Projection.

Breast ultrasound and mammography did not identify any breast masses. Magnetic resonance imaging (MRI) demonstrated cervical occupation (**Figures 1A, B**) and thickening of the rectal wall (**Figures 2A, B**). Gallium-68 fibroblast activation protein inhibitor positron emission tomography-computed tomography (Ga68-FAPI PET/CT) revealed diffuse fibroblast activation in the gastric wall (**Figure 3**). Additionally, 18F-fluorodeoxyglucose positron emission tomography-computed tomography (18F-FDG PET/CT) identified diffuse annular thickening of the rectal wall (**Figure 4**). Notably, 18F-FDG PET/CT did not reveal any lesions in the breast (**Figure 5**).

**Figure 6:**
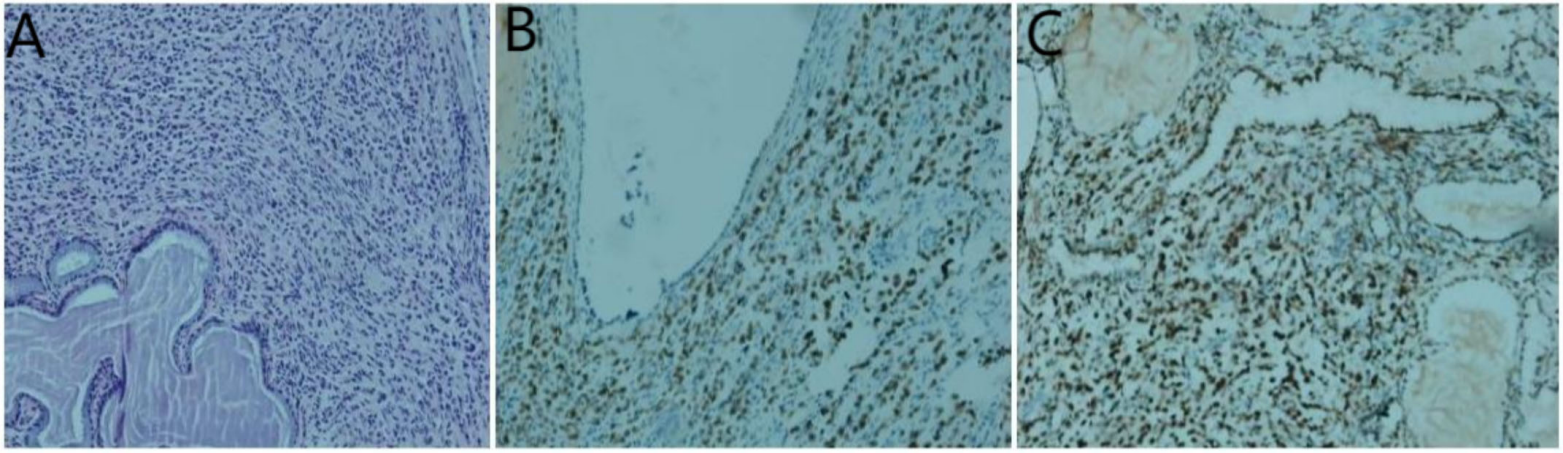
Histological examinations of the cervical specimen. **(A)** Hematoxylin and eosin staining demonstrates the presence of adenocarcinoma within the cervical tissue; **(B)** Immunohistochemistry results reveal positive staining for GATA-3; **(C)** estrogen receptor;(original magnification×200).

Pathological examinations of biopsy samples from the cervix, stomach, and rectum were conducted. The cervical biopsy revealed diffuse infiltration of heterotypic cells within the cervical stroma (**Figure 6A**). Immunohistochemical analysis yielded the following results: AE1/AE3 (+), P63 (-), vimentin (-), CD38 (-), Ki-67 (5% positive cells), CK7 (+), CK20 (-), Synaptophysin (Syn) (-), CDX2 (-), GATA3 (+) (**Figure 6B**), estrogen receptor (ER) (90% strongly positive) (**Figure 6C**), Pax-8 (-), GATA3 (+), E-cadherin (-), HER2 (1+, 60% strongly positive), and TRPS1 (+) (**Figure 6C**). Based on these findings, a mammary origin was strongly suggested, consistent with the diagnosis of poorly differentiated adenocarcinoma.

**Figure 7:**
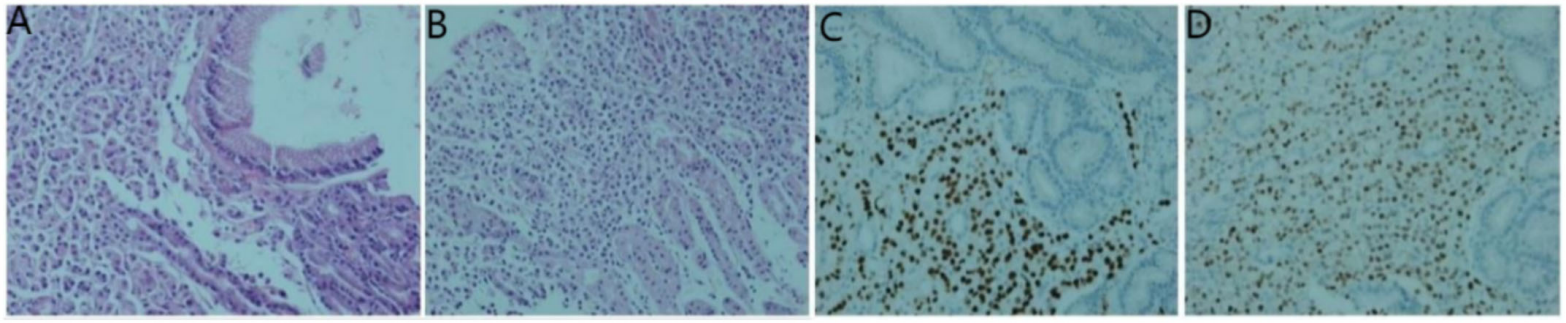
Histological examinations of the gastric specimen. **(A, B)** Hematoxylin and eosin staining demonstrates the presence of adenocarcinoma within the gastric antrum and body tissue; **(C)** Immunohistochemistry results reveal positive staining for GATA-3; **(D)** estrogen receptor.

Secondly, gastroscopy revealed mucosal congestion and edema in the gastric body, along with a bulging lesion approximately 1 cm in size located on the posterior wall of the anterior pyloric area. Pathological examination identified heterotypic cells within the mucosa of both the gastric antrum (**Figure 7A**) and the gastric body (**Figure 7B**). Immunohistochemical analysis yielded the following results: AE1/AE3 (+), CDX2 (-), CD68 (-), Synaptophysin (Syn) (-), CD56 (-), Ki-67 (5%-20% positive cells), CK7 (+), GATA3 (+) (**Figure 7C**), estrogen receptor (ER) (90% strongly positive) (**Figure 7D**), and Pax-8 (-) (**Figure 7D**). Collectively, these findings indicated a diagnosis of poorly differentiated adenocarcinoma, suggesting that the breast should be evaluated as a potential primary site.

**Figure 8:**
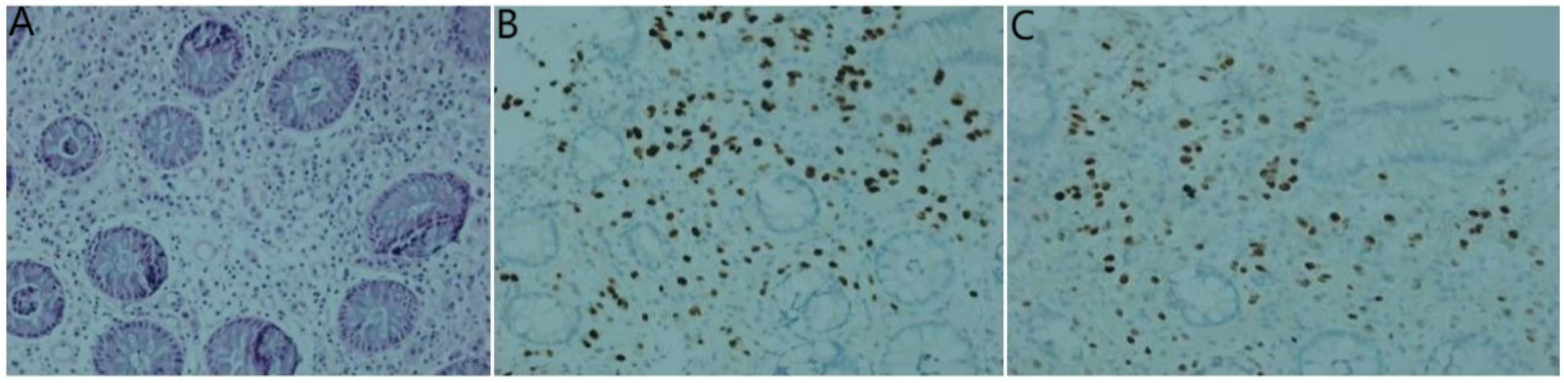
Histological examinations of the rectum specimen. **(A)** Hematoxylin and eosin staining demonstrates the presence of adenocarcinoma within the rectum tissue; **(B)** Immunohistochemistry results reveal positive staining for GATA-3; **(C)** estrogen receptor.

Lastly, enteroscopy revealed rectal mucosal swelling, thickening, stiffness, and lumen stenosis approximately 8 cm from the rectum and anus, making the passage of the scope difficult. Pathological examination identified a small number of heterotypic cells (**Figure 8A**). Immunohistochemical analysis yielded the following results: AE1/AE3 (+), CDX2 (-), CD68 (-), Synaptophysin (Syn) (-), Ki-67 (5% positive cells), CK7 (+), GATA3 (+) (**Figure 8B**), estrogen receptor (ER) (70% strongly positive) (**Figure 8C**), progesterone receptor (PR) (0% positive), HER2 (2+), Mammaglobin (+), and HER2 fluorescence *in situ* hybridization (FISH) (-). Based on these findings, a mammary origin was strongly suggested, consistent with adenocarcinoma.

Histopathological examinations of the cervix, stomach, and rectum revealed stromal adenocarcinoma. Immunohistochemical analysis demonstrated positive staining for GATA-3, whole-cell keratin, and estrogen receptor (ER), supporting the diagnosis of breast cancer metastases to these sites. Additionally, the presence of ER and GATA-3 positivity, along with negative Cudal-type homeobox 2 (CDX2) staining in samples from the cervix, stomach, and rectum, further corroborated the diagnosis of gastrointestinal and cervical metastases originating from breast cancer. However, discrepancies were observed in the expression of hormone receptors, Ki-67, and HER2 among the cervical, gastric, and rectal samples.

No lesions were detected on breast ultrasonography, mammography, or Ga68-FAPI PET/CT. However, the combination of biopsy pathology and immunohistochemical findings confirmed simultaneous metastases to the cervix, stomach, and rectum originating from occult breast cancer. Tumor marker levels were also elevated, with carcinoembryonic antigen (CEA) at 11.88 ng/ml, cancer antigen 15-3 (CA15-3) at 55.96 U/ml, and cancer antigen 125 (CA125) at 63.88 U/ml. Based on these findings, the patient was diagnosed with stage IV occult breast cancer (cTXN0M1) presenting with synchronous metastases to the rectum, stomach, and cervix.

**Figure 9:**
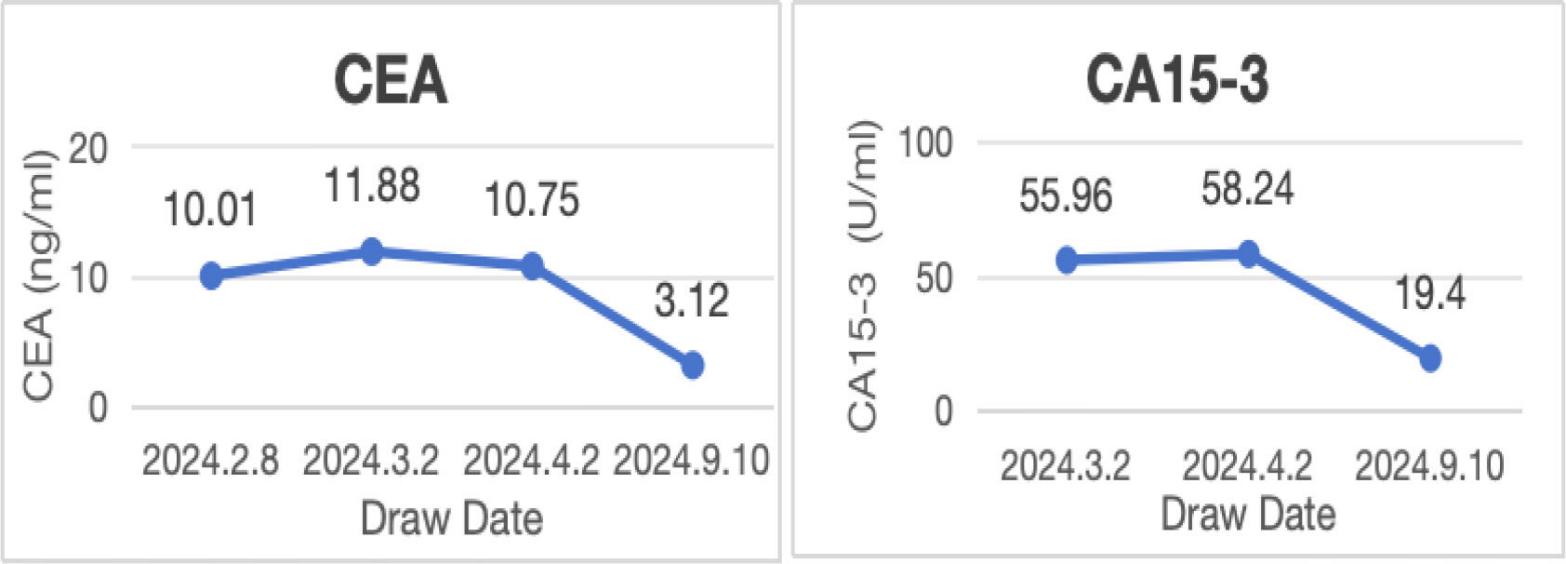
Changes in the levels of tumor markers.

Following the diagnosis, the case was reviewed in a multidisciplinary meeting, which recommended treatment with endocrine therapy combined with CDK4/6 inhibitors and rectal radiotherapy. The patient was prescribed letrozole and dalpiciclib for six months, resulting in a favorable clinical response. She experienced significant relief from severe symptoms, including frequency, urgency, and tenesmus, as well as improvements in stool shape. Follow-up MRI and CT evaluations revealed stable gastric wall lesions, significant reduction of rectal lesions (**Figures 2C, D**), and near-complete resolution of cervical lesions (**Figures 1C, D**) after the combined treatment. Adverse effects were tolerable and primarily included grade II myelosuppression (leukopenia and thrombocytopenia), which improved with symptomatic management. Changes in tumor marker levels during treatment are illustrated in **Figure 9**.

## Discussion

Most patients with OBC are diagnosed at a relatively early stage, often with a favorable prognosis, and can be managed as stage II-III breast cancer (13–15). Key prognostic factors for OBC include axillary lymph node metastasis status, estrogen receptor (ER) status, and the type of local treatment administered (3, 4, 16). Among these, axillary lymph node metastasis is recognized as the most significant adverse prognostic factor (16–18).

Although the incidence of OBC is low, case reports detailing metastases to the bone, liver, lymphatic system, skin, orbit, bone marrow, lung, spleen, or cervix have been published (6–12). To the best of our knowledge, this is the first reported case of occult primary breast cancer with simultaneous metastases to the cervix, stomach, and rectum. While very few cases have documented metastasis to the stomach, rectum, or cervix individually, the rates of gastric and colon metastases in breast cancer patients are estimated as low as 3% and 5%, respectively (19). Simultaneous gastric and colon metastases are exceedingly rare, with only eight cases documented to date (20), and invasive lobular carcinoma is identified as the most common histological subtype associated with gastrointestinal metastasis in breast cancer (21). The likelihood of cervical metastasis in primary breast cancer is similarly rare, with reported rates ranging from 0.8% to 1.7% (22). This rarity can be attributed to the cervix’s small size, dense fibromuscular structure, and access limited to afferent lymphatic vessels, making it an uncommon target for metastatic spread (23). Over 200 cases of breast cancer metastasizing to the uterus have been documented (24). However, a review of case series highlighted that of 27 patients examined, only three presented with isolated cervical metastasis from primary breast cancer, while another 24 cases involved cervical metastasis in conjunction with metastatic disease in other sites (20).

To achieve an accurate diagnosis of OBC, imaging studies for recessive breast cancer remain an area of ongoing investigation. According to the recommendations of the American College of Radiology, MRI, mammography, and ultrasonography should be utilized in patients with suspected OBC who lack evidence of primary breast lesions (25). The diagnostic criteria for OBC include the absence of a detectable mass on both clinical examination and breast X-ray (26). In this case, the patient did not undergo breast MRI; instead, Ga68-FAPI PET/CT was employed, inspired by findings from a relevant report (27). However, the Ga68 PET/CT scan failed to detect any breast lesions in this study.

Pelvic MRI revealed cervical involvement and rectal wall thickening in the patient. 18F-FDG PET/CT and Ga68 PET/CT demonstrated diffuse tumor-associated fibroblast activity in the gastric wall and diffuse annular thickening in the rectal wall. Despite these findings, breast ultrasonography, mammography, and 18F-FDG PET/CT did not identify any breast lesions. Pathological examinations of the cervical, gastric, and rectal tissues were subsequently performed. Histopathological analysis revealed stromal adenocarcinoma in all three sites, and immunohistochemical testing confirmed the presence of markers consistent with breast cancer metastases to the cervix, stomach, and rectum.

For localized OBC, the current National Comprehensive Cancer Network (NCCN) guidelines recommend complete surgical resection of axillary lymph nodes, along with total breast radiotherapy or mastectomy, with or without lymph node radiotherapy (28). A meta-analysis of OBC further supports that breast surgery (BS), including modified radical mastectomy (MRM) and breast-conserving surgery (BCS), combined with radiotherapy, may represent the optimal treatment strategy for patients with localized OBC (29). With advancements in comprehensive therapy, patients with OBC who receive neoadjuvant therapy are generally associated with improved prognoses compared to those who do not undergo neoadjuvant therapy (30–33). Additionally, sentinel lymph node biopsy (SLNB), combined with radiotherapy (RT), may be considered as an alternative to axillary lymph node dissection (ALND) in select breast cancer patients.

To date, limited studies have explored the treatment of extensive metastatic OBC. Similar to the management of non-OBC, systemic therapies—including chemotherapy, radiotherapy, and hormonal therapy—are commonly employed for the treatment of extensive metastatic OBC (34, 35). According to the Guidelines for *Breast Cancer Diagnosis and Treatment* by the China Anti-Cancer Association, patients with hormone receptor-positive, HER2-negative advanced breast cancer, and no evidence of visceral crisis can be treated with endocrine therapy combined with CDK4/6 inhibitors (36). In this case, the patient presented with advanced breast cancer, postmenopausal status, and metastases to the stomach, cervix, and rectum. She was ER-positive, PR-positive, and HER2-negative, and was treated with a regimen of letrozole and dalpiciclib. Clinical trial data have demonstrated that the median progression-free survival is significantly longer in patients receiving dalpiciclib compared to placebo (30.6 months [95% CI: 30.6–not reached] vs. 18.2 months; stratified hazard ratio: 0.51[95% CI 0·38–0·69]) (37). Although the addition of dalpiciclib may increase the incidence of adverse effects such as bone marrow suppression, gastrointestinal reactions, and hepatotoxicity, supportive care and dose adjustments have proven effective in mitigating these toxicities. In this case, after six months of therapy, the patient experienced significant symptom relief and marked reduction in metastatic lesions in the cervix, stomach, and rectum. The treatment regimen was well-tolerated, with manageable side effects limited to bone marrow suppression.

## Conclusion

Non-specific symptoms, such as urinary frequency, urgency, and other atypical clinical manifestations, as observed in this patient, should prompt a high index of suspicion for OBC. Whole-body PET-CT plays a critical role in evaluating potential systemic involvement and identifying underlying primary tumor sites. IHC remains essential for determining the origin of metastatic tumors, even in the absence of detectable lesions in the mammary gland. Furthermore, for patients with ER-positive, HER2-negative OBC presenting at an advanced stage, endocrine therapy combined with CDK4/6 inhibitors represents a promising first-line treatment option.

## Data Availability

The datasets presented in this study can be found in online repositories. The names of the repository/repositories and accession number(s) can be found in the article/Supplementary Material.

## References

[1] Hyuna S Jacques F Rebecca LS Mathieu L Isabelle S Ahmedin J. Global cancer statistics 2020: GLOBOCAN estimates of incidence and mortality worldwide for 36 cancers in 185 countries. CA Cancer J Clin. (2021) 71:209–49. doi: 10.3322/caac.21660, PMID: 33538338

[2] Shaoyuan L Rongshou Z Siwei Z Shaoming W Ru C Kexin S. Global patterns of breast cancer incidence and mortality: a population- based cancer registry data analysis from 2000 to 2020. Cancer Commun (Lond). (2021) 41:1183–1194. doi: 10.1002/cac2.12207, PMID: 34399040 PMC8626596

[3] Mitsuo T Yayoi A Masataka S Masaya H Akiyo Y Gondo N. Occult breast cancer may originate from ectopic breast tissue present in axillary lymph nodes. Breast Cancer Res Treat. (2018) 172:1–7. doi: 10.1007/s10549-018-4898-4, PMID: 30030707

[4] Francesca DC Giuseppe S Francesco F Anna S Giuseppe G. Occult breast cancer in a female with benign lesions. J Cancer Res Ther. (2019) 15:1170–2. doi: 10.4103/jcrt.JCRT_329_17, PMID: 31603129

[5] Hyuna S Jacques F Rebecca LS Mathieu L Isabelle S Ahmedin J. Occult breast cancer: The uncommon presentation of a common disease. Chin Clin Oncol. (2019) 8:S10. doi: 10.21037/cco.2019.01.06, PMID: 30823842

[6] Jue W Geoffrey T Jordan HH Charles E. Occult breast cancer presenting as metastatic adenocarcinoma of unknown primary: clinical presentation, immunohistochemistry, and molecular analysis. Case Rep Oncol. (2012) 5:9–16. doi: 10.1159/000335449, PMID: 22379471 PMC3290025

[7] Ricardo LBC Rubens BC Marilin R Brian JC. Occult breast carcinoma presenting as scalp metastasis. Case Rep Oncol. (2017) 10:992–7. doi: 10.1159/000484346, PMID: 29279704 PMC5731106

[8] Rita PP Júlia F Miguel NB Rui P. Orbital metastasis from an occult breast carcinoma (T0, N1, M1). BMJ Case Rep. (2018) 2018:bcr2017223542. doi: 10.1136/bcr-2017-223542, PMID: 29643136 PMC5898278

[9] Lulu L Jingjing Z Mingtai C Saisai R Haihui L Hao Z. Anemia and thrombocytopenia as initial symptoms of occult breast cancer with bone marrow metastasis: a case report. Medicine. (2017) 96:e8529. doi: 10.1097/MD.0000000000008529, PMID: 29137058 PMC5690751

[10] Ying Z Qiang S Han-yuan H Yi-dong Z Jing-hong G Feng M. Diagnosis and treatment of occult breast cancer: report of 23 cases. Chin J Oncol. (2010) 32:716–8. 21122392

[11] Hyuna S Jacques F Rebecca LS Mathieu L Isabelle S Ahmedin J. Case report: diffuse splenic metastasis of occult breast cancer with incompatible blood group antigenic determinants. Acta Histochem. (2009) 111:343–8. doi: 10.1016/j.acthis.2008.11.015, PMID: 19201455

[12] Emanuela C Jibran A Gabrielle T Svetoslav B Maxim S Nisha L. Unique presentation of occult breast cancer with uterine cervix metastasis. Clin Case Rep. (2019) 7:1573–6. doi: 10.1002/ccr3.2306, PMID: 31428394 PMC6692991

[13] Hyuna S Jacques F Rebecca LS Mathieu L Isabelle S Ahmedin J. Cancer of unknown primary site. Lancet. (2012) 379:1428–35. doi: 10.1016/S0140-6736(11)61178-1, PMID: 22414598

[14] Hyuna S Jacques F Rebecca LS Mathieu L Isabelle S Ahmedin J. Cancer of unknown primary. BMJ. (2020) 371:m4050. doi: 10.1136/bmj.m4050, PMID: 33288500

[15] Mitsuo T Minoru M Hiraku K Hiroaki M Kenji T Masayuki Y. Surgical treatment trends and identification of primary breast tumors after surgery in occult breast cancer: A study based on the Japanese National Clinical Database-Breast Cancer Registry. Breast Cancer. (2022) 29:698–708. doi: 10.1007/s12282-022-01348-y, PMID: 35316446

[16] He M Tang L-C Yu K-D Cao A-Y Shen Z-Z Shao Z-M. Treatment outcomes and unfavorable prognostic factors in patients with occult breast cancer. Eur J Surg Oncol. (2012) 38:1022–8. doi: 10.1016/j.ejso.2012.08.022, PMID: 22959166

[17] Vlastos G Jean M E Mirza A N Mirza N Q Kuerer H M Ames F C. Feasibility of breast preservation in the treatment of occult primary carcinoma presenting with axillary metastases. Ann Surg Oncol. (2001) 8:425–31. doi: 10.1007/s10434-001-0425-6, PMID: 11407517

[18] Qian K Sun W Guo K Zheng X Sun T Chen L. The number and ratio of positive lymph nodes are independent prognostic factors for patients with major salivary gland cancer: Results from the surveillance,epidemiology, and End Results dataset. Eur J Surg Oncol. (2019) 45:1025–32. doi: 10.1016/j.ejso.2018.11.008, PMID: 30472214

[19] Hyuna S Jacques F Rebecca LS Mathieu L Isabelle S Ahmedin J. Gastric and colonic metastases from breast cancer. Am J Dig Dis. (1972) 17:881–6. doi: 10.1007/BF02239526, PMID: 5073677

[20] Sasaki A Masuda S Yoshioka T Saito A Motomura Y. Therapeutic effects of CDK4/6 inhibitors in gastric and colonic metastases from breast cancer: A case report. Cureus. (2024) 16:e52765. doi: 10.7759/cureus.52765, PMID: 38389643 PMC10882214

[21] Ferlicot S Vincent-Salomon A Médioni J Genin P Rosty C Sigal-Zafrani B. Wide metastatic spreading in infiltrating lobular carcinomaof the breast. Eur J Cancer. (2004) 40:336–41. doi: 10.1016/j.ejca.2003.08.007, PMID: 14746850

[22] Hyuna S Jacques F Rebecca LS Mathieu L Isabelle S Ahmedin J. Isolated cervical metastasis of breast cancer: a case report and review of the literature. Gynecol Oncol. (2004) 95:267–9. doi: 10.1016/j.ygyno.2004.07.006, PMID: 15385145

[23] Hyuna S Jacques F Rebecca LS Mathieu L Isabelle S Ahmedin J. Breast cancer metastasising to the uterine cervix. Ulster Med J. (1999) 68:30–2., PMID: 10489810 PMC2449141

[24] Piura B Yanai-Inbar I Rabinovich A Zalmanov S Goldstein J. Abnormal uterine bleeding as a presenting sign of metastases to the uterine corpus, cervix and vagina in a breast cancer patient on tamoxifen therapy. Eur J Obstet Gynecol Reprod Biol. (1999) 83:57–61. doi: 10.1016/S0301-2115(98)00268-1, PMID: 10221611

[25] Hyuna S Jacques F Rebecca LS Mathieu L Isabelle S Ahmedin J. Comparison of contrast-enhanced mammography and contrastenhanced breast MR imaging. Magn Reson Imaging Clin N Am. (2018) 26:259–63. doi: 10.1016/j.mric.2017.12.005, PMID: 29622130

[26] Hyuna S Jacques F Rebecca LS Mathieu L Isabelle S Ahmedin J. Occult primary breast cancer at a comprehensive cancer center. J Surg Res. (2013) 185:684–9. doi: 10.1016/j.jss.2013.06.020, PMID: 23890400 PMC3830668

[27] Hu T Zhang R Zhang B He S Liu L Zou Y. Case report: Uncommon multiple metastases from occult breast cancer revealed by 68Ga-DOTATATE PET/CT. Front Oncol. (2023) 13:1106890. doi: 10.3389/fonc.2023.1106890, PMID: 36910656 PMC9992788

[28] Gradishar W J Anderson B O Abraham J Aft R Agnese D Allison K H. Breast cancer, version 3.2020, NCCN clinical practice guidelines in oncology. JNatl Compr Canc Netw. (2013) 11:753. doi: 10.6004/jnccn.2020.0016, PMID: 32259783

[29] Hyuna S Jacques F Rebecca LS Mathieu L Isabelle S Ahmedin J. Best treatment options for occult breast cancer: A meta-analysis. Front Oncol. (2023) 13:131051232. doi: 10.3389/fonc.2023.1051232, PMID: 37251927 PMC10213692

[30] Hyuna S Jacques F Rebecca LS Mathieu L Isabelle S Ahmedin J. Factors influencing management and outcome in patients with occult breastcancer with axillary lymph node involvement: Analysis of the national cancer database. Ann Surg Oncol. (2017) 24:2907–14. doi: 10.1245/s10434-017-5928-x, PMID: 28766198

[31] Hyuna S Jacques F Rebecca LS Mathieu L Isabelle S Ahmedin J. ASO author reflections: SLNB with RT may be sufficientin occult breast cancer after NAC. Ann Surg Oncol. (2020) 27:1842–3. doi: 10.1245/s10434-020-08345-5, PMID: 32157525

[32] Hyuna S Jacques F Rebecca LS Mathieu L Isabelle S Ahmedin J. Avoiding an axillary lymph node dissection:The benefit of neoadjuvant chemotherapy for occult primary breast cancer. Ann Surg Oncol. (2020) 27:865–6. doi: 10.1245/s10434-020-08939-z, PMID: 32725524 PMC7680299

[33] Hyuna S Jacques F Rebecca LS Mathieu L Isabelle S Ahmedin J. Micrometastases or isolatedtumor cells and theoutcome of breast cancer. NEnglJMed. (2009) 36:653–63. doi: 10.1056/NEJMoa0904832, PMID: 19675329

[34] Hyuna S Jacques F Rebecca LS Mathieu L Isabelle S Ahmedin J. Breast cancer with synchronous massive metastasis in the uterine cervix: a case report and review of the literature. Arch Gynecol Obstet. (2010) 281:769–73. doi: 10.1007/s00404-009-1264-0, PMID: 19876639

[35] Hyuna S Jacques F Rebecca LS Mathieu L Isabelle S Ahmedin J. Application of neoadjuvant chemotherapy in occult breast cancer: five case reports. Medicine. (2017) 96:e8200. doi: 10.1097/MD.0000000000008200, PMID: 28984771 PMC5738007

[36] Hyuna S Jacques F Rebecca LS Mathieu L Isabelle S Ahmedin J. Guidelines for breast cancer diagnosis and treatment by China Anti-cancer Association (2024 edition). China Oncol. (2023) 33:1092–187. doi: 10.19401/j.cnki.1007-3639.2023.12.004

[37] Hyuna S Jacques F Rebecca LS Mathieu L Isabelle S Ahmedin J. Dalpiciclib plus letrozole or anastrozole versus placebo plus letrozole or anastrozole as first-line treatment in patients with hormone receptor-positive, HER2-negative advanced breast cancer (DAWNA-2): a multicentre, randomised, double-blind, placebo-controlled, phase 3 trial. Lancet Oncol. (2023) 24:646–57. doi: 10.1016/S1470-2045(23)00172-9, PMID: 37182538

